# Unveiling Synergistic Hydration in a Multi-Waste Binder: Co-Processing Electrolytic Manganese Residue and Red Mud with Steel Slag for Enhanced Performance

**DOI:** 10.3390/ma18204711

**Published:** 2025-10-14

**Authors:** Yingchun Sun, Xinglan Cui, Xiaobin Gu, Xinyue Shi, Hongxia Li, Lei Wang

**Affiliations:** 1 National Engineering Research Center for Environment-Friendly Metallurgy in Producing Premium Non-Ferrous Metals, China GRINM Group Co., Ltd., Beijing 101407, China; 15966001661@163.com (Y.S.); shixinyue@grinm.com (X.S.); lihongxia@grinm.com (H.L.); wanglei@grinm.com (L.W.); 2 GRINM Resources and Environment Tech. Co., Ltd., Beijing 101407, China; 3 General Research Institute for Nonferrous Metals, Beijing 100088, China; 4 National Engineering Research Center for Environment-Friendly Metallurgy in Producing Premium Non-Ferrous Metals, Beijing 101407, China; 5 Beijing Engineering Research Center of Strategic Nonferrous Metals Green Manufacturing Technology, Beijing 101407, China; 6 Key Laboratory of Comprehensive and Highly Efficient Utilization of Salt Lake Resources, Qinghai Institute of Salt Lakes, Chinese Academy of Sciences, Xining 810008, China; guxb@isl.ac.cn

**Keywords:** EMR, RM, waste-based binder, sulfate-based activation, resource utilization

## Abstract

In response to the pressing environmental challenges posed by electrolytic manganese residue (EMR) and red mud (RM), this study proposes an innovative cementitious material technology for the synergistic co-utilization of these industrial wastes. By employing steel slag (SS) as a calcium-rich skeleton, the system effectively immobilizes sulfates from EMR and alkalinity from RM, converting hazardous wastes into value-added construction materials. Through orthogonal experimentation, an optimal mix proportion was established—30% RM, 20% EMR, and 50% SS at a water-to-binder ratio of 0.28—which achieved a 28-day compressive strength of 20.40 MPa, meeting relevant industry standards for auxiliary cementitious materials. Microstructural analysis unveiled a multi-stage alkali-sulfate synergistic activation mechanism: (1) the high alkalinity derived from RM rapidly activates the dissolution of aluminosilicate phases in both SS and EMR; (2) sulfate ions released from EMR promote extensive formation of ettringite (AFt), enhancing early-age structural integrity; and (3) calcium ions from SS facilitate the development of a dense C-S-H gel matrix, which serves as the primary binding phase. More profoundly, this process exemplifies a self-stabilizing waste-to-resource conversion mechanism, whereby harmful constituents (sulfates and free alkalis) are constructively incorporated into stable hydration products. This work not only elucidates a coherent scientific framework for the safe and efficient reclamation of multi-source solid wastes, but also demonstrates a scalable and ecologically viable pathway for million-ton-scale valorization of EMR and RM. Furthermore, it presents feasibility insights for the application of high-dosage steel slag-based material systems, thereby unifying significant environmental and economic advantages.

## 1. Introduction

As China accelerates its industrial development, the annual generation of large-volume industrial solid wastes—such as red mud, electrolytic manganese residues, and steel slag—continues to rise. With accumulated stockpiles surpassing 6 billion metric tons and a comprehensive utilization rate of less than 55%, these materials not only impose significant environmental pressure but also squander their precious resource value [1]. Currently, the national level places great emphasis on the resource utilization of solid waste, the call for action emphasizes the need to accelerate the development of integrated utilization capabilities for complex and difficult-to-manage solid waste, with a focus on expanding its high-value applications in areas such as green building materials, road base materials, and soil remediation.

Electrolytic Manganese Residue (EMR) is a hazardous solid waste generated after solid–liquid separation during the filter press stage of electrolytic manganese metal production. Based on typical manganese carbonate ore grades, approximately 8–12 tons of manganese residue are generated per ton of manganese metal produced [2]. Hazardous ions in EMR are highly susceptible to migrating into groundwater and soil under rainwater erosion, leading to a series of environmental pollution issues [3]. Fresh EMR exhibits high environmental mobility and has a moisture content of about 30% [4]. Its primary components include gypsum and quartz, along with soluble harmful substances such as manganese sulfate and ammonia nitrogen [5]. Currently, open-air slag reservoirs serve as the dominant disposal method [6], where prolonged stockpiling accelerates contaminant migration, intensifying risks to surrounding aquifers and terrestrial ecosystems [7]. China is the world’s largest producer of electrolytic manganese metal (EMM), with an estimated production of 1.15 million metric tons, according to industry reports in 2023 [8]. This exponential growth of residual waste now critically constrains sustainable development within the China’s electrolytic manganese sector [9]. Consequently, the development of effective treatment strategies for EMR is imperative to mitigate its escalating environmental burden [10].

Red mud (RM), an industrial residue from alumina extraction, derives its name from iron oxide-induced reddish coloration [10]. It is classified as a typical bulk industrial solid waste in the aluminum production sector [11]. Depending on the process technology and ore type, the production of 1.0 ton of alumina typically generates 1–2 tons of red mud [12]. Compared to developed countries, China’s comprehensive utilization rate of red mud is significantly lower, standing at merely 4% [13]. This has led to the accumulation of over 3.5 billion tons of red mud stored within large storage dams located across the country, with the stockpile currently increasing by approximately 100 million tons annually. The vast open-air stockpiling of red mud poses multiple threats to the surrounding ecological environment: on one hand, red mud disposal occupies substantial valuable land resources; on the other hand, its strong alkalinity and high salt content can cause severe pollution to soil, groundwater, and atmospheric environments [14]. Furthermore, excessive stockpiling could also lead to secondary environmental disasters, such as dam failures, which pose substantial risks to ecological security, as well as human life and property. For instance, the dam collapse accident at the Ajkai Timfoldgyar alumina plant in Hungary in 2011 resulted in multiple casualties and the complete collapse of the nearby river ecosystem [15]. Consequently, how to safely, efficiently, and scalably dispose of and utilize red mud as a resource, in order to minimize its potential threats to the ecological environment and human health, has become a critical scientific and engineering challenge urgently needing resolution.

Steel Slag (SS) is one of the primary solid wastes discharged during the steelmaking process, mainly including converter slag, electric furnace slag, and refining slag. Its output accounts for approximately 15% to 20% of crude steel production [16]. In recent years, with the rapid development of China’s iron and steel industry, the accumulated stockpiles of steel slag have been steadily increasing. In recent years, with the rapid development of China’s iron and steel industry, the accumulated stockpiles of steel slag have been steadily accumulating. Furthermore, long-term storage of steel slag makes it susceptible to weathering, causing it to fragment into fine particles. These respirable particulate matters can disperse under wind action, resulting in atmospheric pollution that has severe impacts on the health of nearby residents and the quality of the ecological environment [17]. However, steel slag is not “waste” in the conventional sense. The active C_2_S and C_3_S components within steel slag exhibit cementitious properties, which can be harnessed as they offer high strength, stability, and durability [18].

SS, EMR, and RM differ in their applications within the building materials industry. Research revealed that steel slag contained active minerals similar to cement clinker, such as C_2_S, C_3_S, and C_2_F, and proper activation could develop their potential activity. Electrolytic manganese residue contained gypsum minerals accounting for over 30%, which could act as sulfate activators. The Bayer process red mud had an alkali content exceeding 10%, with soluble alkali predominantly present. Some chemically combined alkalis can be converted into soluble alkalis through calcination treatment [19]. Upon dissolution in water, they create an alkaline environment and can serve as alkali activators. This study adopts EMR, RM, and SS as raw materials for preparing compo-site cementitious material [20]. Through systematic optimization of the mix ratios involving steel slag, red mud, and electrolytic manganese residue, the research delves into the hydration products and mechanisms, with a particular focus on the activation of steel slag using red mud and electrolytic manganese residue. The findings offer foundational insights for large-scale utilization of red mud and electrolytic manganese residue in China.

## 2. Materials and Methods

### 2.1. Raw Materials Characterization

**Figure 1:**
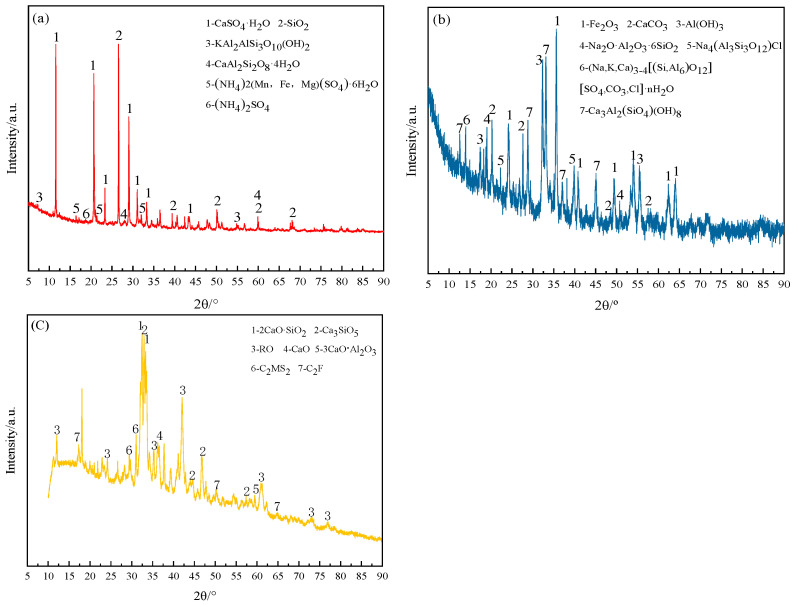
Mineralogical phases of raw materials ((**a**) is EMR, (**b**) is RM, (**c**) is SS).

This study employed RM, EMR, and SS as the primary materials. Their chemical composition was characterized using Panalytical Axios X-ray fluorescence (XRF) spectrometry (PANalytical B.V., Almelo, The Netherlands), with results detailed in Table 1. Mineralogical analysis conducted with the Rigaku SmartLab SE (Rigaku Corporation, Akishima, Tokyo, Japan), a premier multipurpose X-ray diffraction system equipped with intelligent guidance software, unveiled the mineral compositions of the raw materials, as depicted in Figure 1.

As indicated in Table 1, the primary chemical compositions of electrolytic manganese residue include SO_3_, CaO, SiO_2_, etc.; for red mud, Fe_2_O_3_, CaO, Al_2_O_3_, SiO_2_, etc.; and for steel slag, Fe_2_O_3_, CaO, Al_2_O_3_, SiO_2_, etc. As shown in Figure 1, the dominant mineral phases in electrolytic manganese residue are gypsum and silica; in red mud, they are hematite (Fe_2_O_3_), potassium feldspar, and dolomite; while in steel slag, the primary phases are dicalcium silicate (C_2_S), tricalcium silicate (C_3_S), RO phase (solid solution of divalent oxides), and free CaO. The experimental protocol for the toxicity leaching of raw materials is detailed in Table 2, as outlined in the toxicity leaching experiment report. This report encompasses specific parameters such as concentrations and time frames, alongside the outcomes of the experiments. Toxicity leaching is conducted according to HJ/T229-2007 [21] sulfuric acid nitric acid method standard.

### 2.2. Experimental Procedures

By employing orthogonal experimental designs with three factors and four levels, the research seeks to pinpoint key influencing variables and ascertain the optimal mix proportion. Studies employing L16 orthogonal tables have systematically evaluated the effects of various factors on concrete strength, as demonstrated in similar research. The specific experimental ratios are detailed in Table 3, Table 4 and Table 5, which provide a comprehensive overview of the material proportions used in the study.

**Figure 2:**
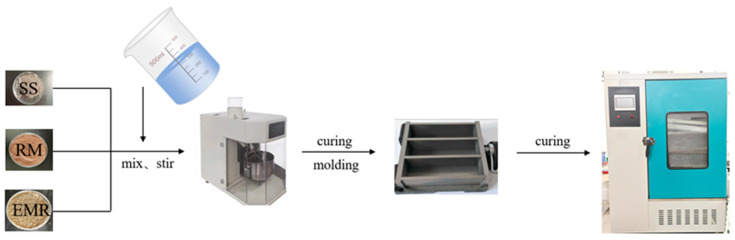
Cementitious Material Process Flow.

The preparation process begins with weighing the raw cementitious materials in the required proportions. Following thorough blending, the mixture is transferred to a cement mortar mixer (Tianjin Xichang Instrument Technology Co., Ltd., Tianjin, China). An automated program initiates agitation to ensure homogeneity within the resulting slurry. This slurry is then poured into steel molds (40 mm × 40 mm × 160 mm), subjected to jolting compaction, and surface-leveled with a trowel. Finally, the samples are sealed using plastic film to minimize moisture evaporation while preventing environmental contamination [22]. To simulate actual service conditions, the curing environment is maintained at 29 °C and 90% relative humidity [23]. Following a curing period of 3 days, specimens that have reached initial hardening are demolded and undergo compressive strength evaluation in accordance with established testing protocols. Specimens designated for prolonged analysis continue curing until designated ages prior to comprehensive performance evaluation. Post-strength-test samples are immersed in ethanol to arrest ongoing hydration reactions. This allows for multi-technique characterization—via X-ray diffraction (XRD), Fourier-transform infrared spectroscopy (FTIR), thermogravimetric analysis (TGA), and scanning electron microscopy (SEM)—of samples collected at varied hydration stages. These analyses facilitate investigation into hydration kinetics, reaction products, and underlying mechanisms. Figure 2 illustrates the specific production process of the red mud-based electrolytic manganese residue cementitious material [24].

Accurate detection of water content is essential for the effective synthesis of cementitious binders derived from solid waste, as it ensures the quality and performance of the final product. Water serves two main functions: maintaining the plasticity and fluidity of the cementitious material, and providing sufficient moisture for the hydration reaction. Specifically, water-to-binder ratios of 0.28, 0.30, 0.32, 0.34, and 0.36 have been found to influence the strength and durability of concrete, with lower ratios typically correlating with higher compressive strength. For instance, Reference [25] details how different water-to-binder ratios were used to establish a functional relationship between the ratio and the measured compressive strength of concrete at various ages, demonstrating that a decrease in water-to-binder ratio leads to an increase in compressive strength. Based on previous practical experience and recent research, a preliminary ratio of RM:SS:EMR = 2:2:1 was adopted to maximize the synergistic activation effect. Table 3 presents the experimental design for this study.

To further optimize the mix proportions of other raw materials in the solid waste-based cementitious material, an orthogonal experimental design with three factors and four levels was established, utilizing red mud dosage, electrolytic manganese residue dosage, and water-to-binder ratio as the three factors. This design aims to investigate the influence of different factors on the properties of the solid waste-based cementitious material. The orthogonal experimental factor table and the orthogonal array are presented in the table below. The raw materials for the cementitious material system in this study solely consist of RM, EMR, and SS (with red mud and electrolytic manganese residue dosages expressed as a percentage of the total cementitious material). The specific experimental mix proportions are detailed in Table 5 and Table 6.

### 2.3. Testing and Characterization Methods

#### 2.3.1. Strength Testing

In this study, TYE-300D cement mortar bending and compression tester (Wuxi Jianyi Instrument & Machinery Co., Ltd., Wuxi, China) was used to test the compressive strength of all test sample [26]. The compressive strength test was conducted at a loading rate of 0.6 kN/s in accordance with GB/T 17671-1999 [27] of China. Each group of test blocks each time select 2 samples for testing; the system will automatically process the data, calculate, and save the average value.

#### 2.3.2. Microscopic Characterization

The elemental compositions of the raw materials were determined by X-ray fluorescence (XRF) spectrometry using a Panalytical Axios sequential spectrometer. Phase evolution of the starting materials and hydration products was investigated by X-ray diffraction (XRD) performed on a Rigaku-RA high-power rotating-anode diffractometer (12 kW) with Cu Kα radiation, with a step size of 0.02°, a 2θ scanning range of 10–90°, a scan rate of 5 ° min^−1^, an accelerating voltage of 40 kV and a tube current of 150 mA [28]. The microstructure and spatial distribution of hydration products in the hardened pastes were examined by field-emission scanning electron microscopy (FE-SEM) using a Zeiss SUPRA 55 microscope (Carl Zeiss, Oberkochen, Germany). Molecular structures and chemical bonds were analyzed by Fourier-transform infrared (FTIR) spectroscopy on a Shimadzu FTIR-8400s spectrometer (Shimadzu Corporation, Kyoto, Japan), enabling compound identification through characteristic wavenumbers [29]. Differential scanning calorimetry (DSC) was performed with a Netzsch STA 409C/CD instrument (Netzsch-Gerätebau GmbH, Selb, Germany) under an argon atmosphere from 25 to 1000 °C at a heating rate of 10 °C min^−1^; the resulting endothermic and exothermic peaks, in conjunction with XRD and FTIR data, were employed to corroborate the assignment of reaction products [30].

## 3. Results

### 3.1. Influence of Raw Material Ratio on the Properties of Cementitious Materials

**Figure 3:**
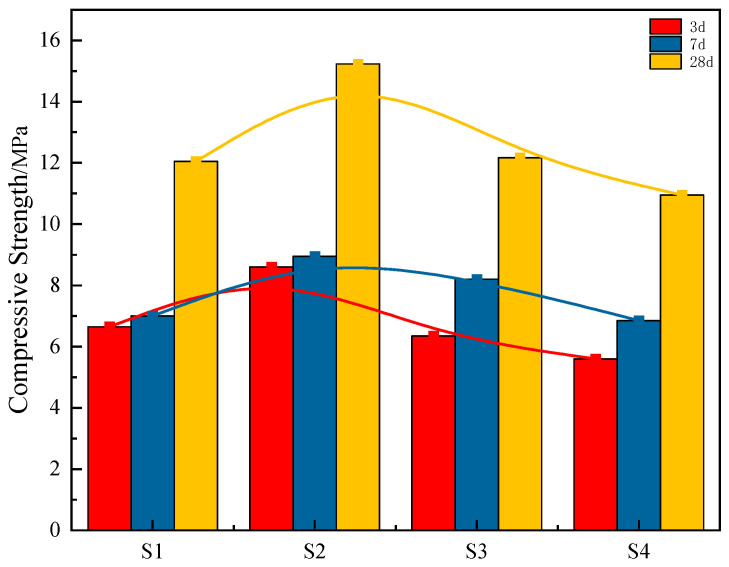
Compressive Strength Test Results of Different Raw Material Ratios.

The compressive strengths of S1–S4 are presented in Figure 3. As shown in the figure, the compressive strength of the S1 group specimens is significantly lower, which can be attributed to the reduced steel slag content in the specimens. Although steel slag exhibits low reactivity, it is rich in divalent metal cations. After hydration, it rapidly increases the alkalinity of the system and generates a substantial amount of Ca^2+^, thereby promoting the dissociation of silicon (aluminum) oxyanions tetrahedrons in the red mud [31]. Consequently, with a low steel slag content, the system suffers from insufficient alkalinity, lower levels of Ca^2+^ and OH^−^, resulting in slower hydration reactions and, consequently, lower strength. Elevated steel slag content correlates with increased Ca(OH)_2_ formation during hydration. Under the activation effects of electrolytic manganese residue and the alkaline environment, the silicate (aluminate) tetrahedrons in the red mud depolymerize and then repolymerize, absorbing Ca^2+^ released from steel slag hydration. This process produces large quantities of C-S-H gel and AFt, leading to a significant increase in the strength of the specimens across all ages. However, with further increases in steel slag content, the block body strength at all ages shows a decreasing trend [32]. This is due to the reduced proportion of red mud in the mixture, diminishing the amount of reactive silicon (aluminum) oxygen tetrahedrons, and therefore, fewer hydration products are formed in the specimens.

### 3.2. Influence of Water-Glue Ratio on Properties of Cementitious Materials

For the preparation of solid waste-based cementitious materials, determining water content serves two primary functions: first, maintaining the plasticity and fluidity of the cementitious material, and second, providing sufficient moisture for the hydration reaction. Insufficient water content results in incomplete hydration reactions, reducing the compressive strength of test blocks; excessive water content may impede sample formation, leading to decreased compressive strength [33]. Water-glue ratios were set at 0.28, 0.30, 0.32, 0.34, and 0.36, respectively, with other mix proportions tentatively established based on previous practical experience as RM:SS:EMR = 2:2:1. This approach aligns with research findings that indicate the use of waste materials can contribute to the strength development of concrete mixtures, as shown in studies [34]. Additionally, the chosen water-glue ratios are in line with those used in other studies that have shown to enhance the compressive strength of concrete [35]. Detailed experimental procedures can be referenced in Table 3.

**Figure 4:**
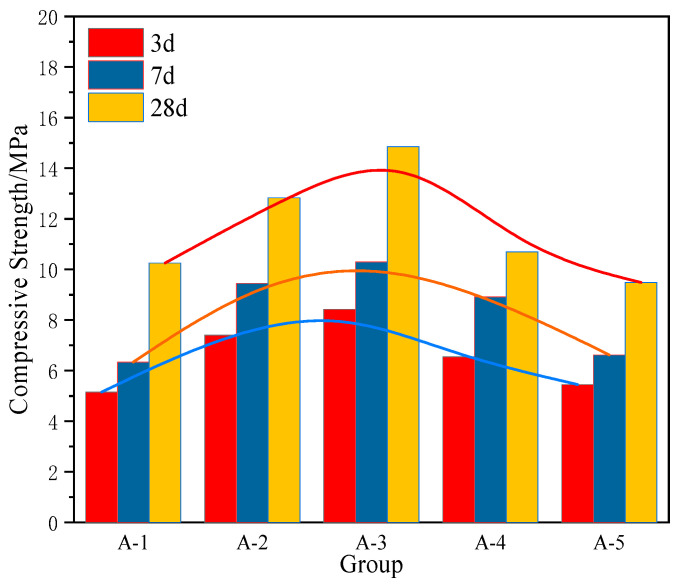
Compressive Strength Test Results of Different Water-Glue Ratios.

In this study, systematic analyses were conducted on the compressive strength of solid waste-based cementitious materials with varying water-glue ratios (ranging from 0.28 to 0.36). Based on the results shown in Figure 4, with increasing water-glue ratio, the compressive strength of the material exhibits a specific trend of variation. Specifically, during the 3-day, 7-day, and 28-day testing periods, the compressive strength initially increases and then decreases as the water-glue ratio increases. This phenomenon may be closely related to the sufficiency of hydration reactions and the formation of internal structure in the material, which are significantly influenced by the water-glue ratio. A lower ratio (e.g., 0.28 and 0.30) may result in incomplete hydration reactions, thereby adversely affecting compressive strength. Conversely, a higher ratio (e.g., 0.34 and 0.36), while potentially enhancing compressive strength in the short term, may compromise the structural stability of the material over time, ultimately impairing its long-term compressive performance and durability considerations. This conforms to the theoretical framework on how strength is affected by moisture [36]. Based on compressive strength measurements across all groups, in the subsequent orthogonal experiment for further optimizing the mix proportion of the binder materials, the water-glue ratio was set within the range of 0.28 to 0.34.

### 3.3. Orthogonal Experimental Analysis of Cementitious Materials

**Figure 5:**
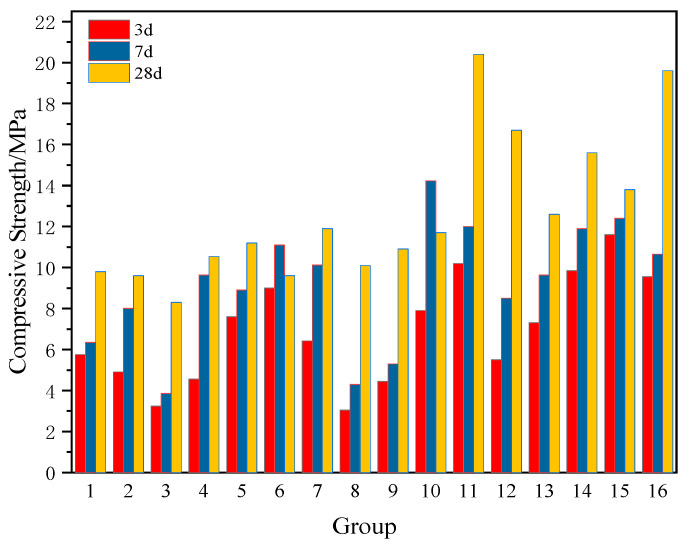
Test Results of Compression Resistance by Orthogonal Experiment.

As shown in Figure 5, at 3 days, all specimens exhibited compressive strengths greater than 3 MPa. At 7 days, specimens SZ-5, SZ-11, SZ-14, SZ-15, and SZ-16 demonstrated strengths exceeding 10 MPa, while SZ-3, SZ-8, and SZ-9 showed relatively lower values. By 28 days, all specimens except SZ-4 achieved strengths above 9 MPa, among which SZ-11 reached the maximum value of 20.40 MPa.

#### 3.3.1. Orthogonal Experiment Visual Analysis of Cementitious Materials

The advantages of the intuitive analysis method lie in its straightforwardness and ease of application, as well as its visual clarity [37]. However, this approach cannot effectively differentiate between data fluctuations and experimental errors. Moreover, it fails to assess the significance of factors’ influence on experimental results. Intuitive analysis calculates average values at each level to determine the range and subsequently assesses the extent to which factors influence experimental results by comparing these averages. Comparing the magnitude of the range values. According to Table 6, R_RM_ exceeds R_WGR_, which in turn surpasses R_EMR_, indicating that red mud exerts the greatest impact on the compressive strength among the three factors in this experiment. By examining the mean value of each factor, we can tentatively conclude that the optimal factor level combination for this experiment is factor 1 at level 4, factor 2 at level 3, and factor 3 at level 1. Table 7 shows the range analysis table.

#### 3.3.2. Analysis of Variance Method of Cementitious Material

Analysis of Variance (ANOVA) can be used to assess experimental error magnitude, reveal experimental precision, and determine the order of influence of various factors and their interaction effects on experimental indicators [38]. In addition, ANOVA identifies factors significantly affecting experimental outcomes and guides the selection of optimal levels for strict control. For factors with non-significant effects, appropriate levels can be determined based on specific circumstances.

The experimental results, as indicated in Table 8, demonstrate that red mud plays a crucial role in enhancing the outcomes. Specifically, the optimal mix proportion that yields the highest compressive strength consists of 30% red mud, 20% electrolytic manganese residue, and a water-glue ratio of 0.28.

#### 3.3.3. Verification Experiment of Gel Material

Verification experiments were conducted based on the orthogonal experimental results. Three sets of tests were performed using the optimal ingredient ratio determined above to validate the accuracy of the orthogonal experiment. The outcomes of these experiments are presented in Table 9.

As shown in Table 9: The average 3-day compressive strength of Specimen Group 1.2.3 is 10.27 MPa, with a discrepancy of 0.07 MPa from the orthogonal experiment results. The 7-day average compressive strength reaches 11.95 MPa, deviating by 0.05 MPa from the orthogonal experiment. The 28-day average compressive strength measures 20.48 MPa, showing a 0.08 MPa variance from orthogonal experimental data. All three measurement sets exhibit discrepancies under 1% (less than 0.08 MPa) compared to orthogonal experiment outcomes, indicating consistent results.

#### 3.3.4. Comparative Experiment of Gel Material

Test blocks prepared using Type 42.5 cement served as the control group. Their compressive strengths were measured at 3 d, 7 d, and 28 d. The mix proportion for the cement control group was a sand-cement ratio of 1:1 and a water content of 23%. The compressive strengths at 3 d, 7 d, and 28 d were determined according to the above proportions. Table 10 presents the comparison results between the cement control group and the solid waste-based cementitious material incorporating red mud-electrolytic manganese residue-steel slag.

As shown in Table 10, the 28-day compressive strength of the Red Mud-Electrolytic Manganese Residue-Steel Slag composite solid waste cementitious material (RMSS-CM) is 20.4 MPa, meeting the standard specification for blended hydraulic cements (ASTM C595). Although RMSS-CM exhibits a slight performance gap compared to conventional cement-based binders in some traditional aspects, it possesses significant advantages in cost effectiveness, environmental sustainability, and renewability. Overall, RMSS-CM demonstrates considerable development potential as a viable alternative to traditional cement-based binders in certain applications.

## 4. Discussion

In order to elucidate the strength development mechanism and hydration behavior of the optimized SZ-11 blend system, this study employed a suite of advanced analytical techniques to comprehensively characterize the sample’s phase composition, morphological microstructure, structural evolution, and thermal stability at various curing ages. XRD was employed for phase identification and semi-quantitative analysis of the hydration products, facilitating the tracking of crystalline mineral content. formation and transformation throughout the hydration process. FTIR was utilized to analyze the vibrational characteristics of functional groups in hydration products, uncovering changes in chemical bonding and hydration levels. SEM was utilized to monitor the microstructural changes, enabling a detailed examination of the morphology and dispersion of hydration products. This analysis is instrumental in establishing the relationship between these hydration products and the mechanical strength of the material. TGA was performed to investigate the characteristics of mass loss during hydration, thereby assessing the thermal stability of the hydration products and the variation in bound water content. Consequently, this integrated approach established the mechanistic interplay between hydration kinetics and strength evolution in the SZ-11 system across discrete curing regimes.

### 4.1. XRD Analysis

**Figure 6:**
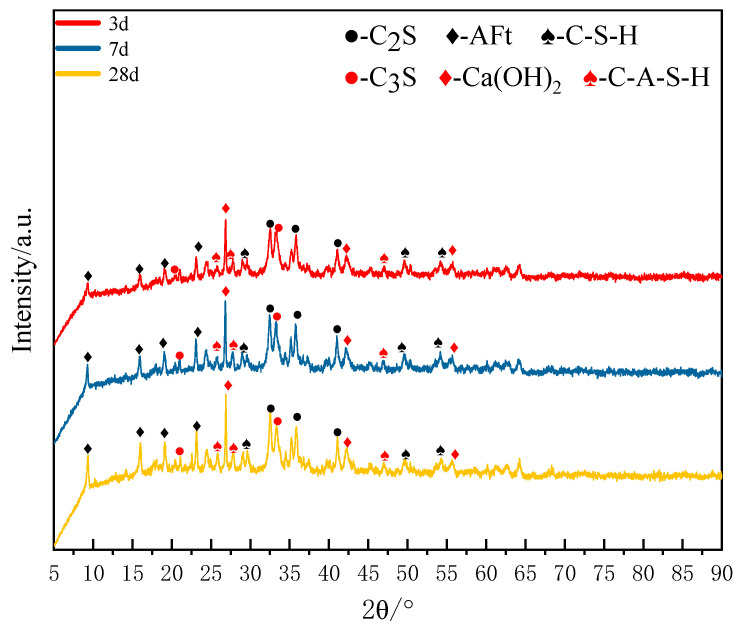
XRD of SZ-11 at 3 d, 7 d, and 28 d.

The XRD patterns of SZ-11 hydrated for 3, 7 and 28 days are presented in Figure 6.

As shown in Figure 6, the primary hydration products of the SZ-11 specimen comprise a series of zeolite-like crystalline phases. The inclusion of amorphous or weakly crystalline calcium silicate hydrates (C-S-H) significantly enhances the early-stage strength of cement-based systems. The layered structure of C-S-H forms a dense microscopic skeleton, which contributes to the rapid development of strength. Aft (chemical formula Ca_6_Al_2_(SO_4_)_3_(OH)_12_·26H_2_O) exists as slender acicular crystals, which further improve the long-term strength of the specimen by filling capillary pores and generating micro-expansion effects. Additionally, the calcium aluminate silicate hydrate (C-A-S-H) phase is detected, exhibiting structural features that integrate the cross-linked characteristics of both C-S-H and C-A-S-H. Its three-dimensional network effectively confines Al^3+^ and Si^4+^ ions, thereby yielding a more compact structure. The XRD pattern of the raw materials simultaneously reveals diffraction peaks of dicalcium silicate (C_2_S) and tricalcium silicate (C_3_S). As pivotal mineral phases in Portland cement clinker, these compounds provide abundant sources of Ca^2+^ and SiO_4_^4−^ ions. Under the action of the alkaline activator, they rapidly dissociate, dissolve, and participate in early-stage hydration reactions. By the 3 d, Characteristic diffraction peaks of Aft were clearly observed within the SZ-11 system. This indicates that the ion concentrations of SO_4_^2−^ and AlO_2_^−^ in the SS-EMR-RM alkali-activated system had reached the supersaturation threshold, thereby promoting the rapid nucleation and growth of Aft. As the hydration age continuously extended from 3 days to 7 days and then to 28 days, the intensity of AFt’s diffraction peaks exhibited a significant increasing trend. This signifies a continuous increase in the crystal size of Aft, an improvement in its crystallinity, and a corresponding rise in the AFt content within the system. This result provides indirect confirmation that continuously supplied ions such as Ca^2+^, Al(OH)_4_^−^, and SO_4_^2−^ diffuse to the surface of Aft crystals and participate in crystal growth via a dissolution-precipitation mechanism, consequently strengthening the microstructure of the matrix [39]. SS and RM-bearing reactive CaO, SiO_2_, Al_2_O_3_-promptly liberate significant Ca^2+^/OH^−^ ions with trace alkali cations (Na^+^, K^+^) in alkaline media. This rapid ion release elevates pore solution pH > 12.5, establishing critical conditions for C-S-H gel nucleation and Ca(OH)_2_ crystallization. Relevant chemical equations for this system are as follows [40,41,42]:

C_2_S + H_2_O → C-S-H + Ca(OH)_2_, (1)

C_3_S + H_2_O → C-S-H + Ca(OH)_2_, (2)

SiO_2_ + OH^−^ + H_2_O → [H_3_SiO_4_]^−^ (3)

AlO_2_^−^ + OH^−^ + H_2_O → [H_3_AlO_4_]^2−^ (4)

[H_3_AlO_4_]^2−^ + [H_3_SiO_4_]^−^ + Ca^2+^ → C-A-S-H (5)

6Ca^2+^ + 2Al(OH)_4_^−^ + 3SO_4_^2−^ + 4OH^−^ + 24H_2_O ⟶ Ca_6_Al_2_(SO_4_)_3_(OH)_12_⋅26H_2_O (6)

In general, the early stage (3 d) is primarily driven by dissolution–nucleation, with the initial formation of AFt and C-S-H. The mid-term stage (7 d) shows accelerated reactions and refinement of the gel structure. In the later stage (28 d), the reaction has approached completion, characterized by the full development of AFt crystals, highly polymerized C-S-H, and microstructural densification.

### 4.2. FTIR Analysis

**Figure 7:**
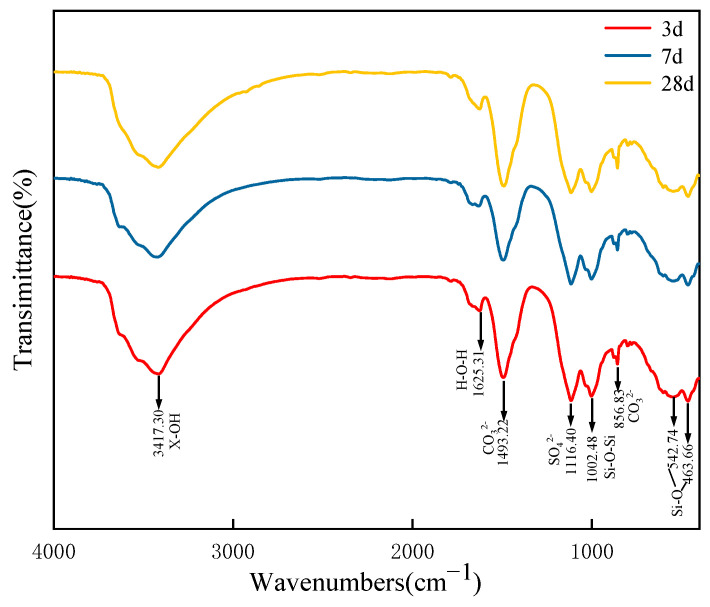
Spectra of road base mix at different curing periods.

In this study, FTIR spectra were obtained for optimally blended SZ-11 specimens at various hydration stages (3, 7, and 28 days), capturing the molecular vibrational information over the wavenumber range of 4000–500 cm^−1^. These spectra, which are crucial for understanding the chemical composition and structural changes during hydration, are detailed in Figure 7. Characteristic absorption peaks were observed at 3417, 1625, 1493, 1116, 1002, 856, 542 and 463 cm^−1^ for all samples.

As shown in Figure 7, an absorption peak appeared at 3417 cm^−1^, primarily attributed to the asymmetric stretching vibration of (X-OH) [43]. The bending vibration of H-O-H in C-S-H gel is mainly characterized by the absorption peak at 1625 cm^−1^ [44]. These two absorption peaks collectively demonstrate that the hydration reaction process results in substantial substances rich in water of crystallization. In the infrared spectroscopy results of the 3-day sample, the SO_4_^2−^ stretching vibration of AFt was observed, characterized by an absorption peak at 1116 cm^−1^ [45]. This indicates the formation of AFt in the specimen at 3 days, a result consistent with the XRD analysis. The absorption peak at 1493 cm^−1^ is associated with the vibration of CO_3_^2−^, while the peak at 856 cm^−1^ corresponds to the bending vibration of CO_3_^2−^. These sharp peaks arise from the reaction between atmospheric carbon dioxide and alkali components in geopolymer, combined with asymmetric stretching of CO_3_^2−^ [46]. The absorption peaks observed at 542 cm^−1^ and 463 cm^−1^ are attributed to vibrations of the Si-O bond.

In addition, by comparing the absorption peak at 1116 cm^−1^ for specimens of different ages, it was observed that this peak sharpen progressively (or gradually) with increasing age. This result indicates that the degree of hydration reaction increases gradually with age, leading to a continuous increase in the degree of polymerization of the AFt (or resulting AFt) produced by the hydration. The absorption peak near 1002 cm^−1^ corresponds to the asymmetric stretching vibrations of Si-O-Si and Si-O-Al bonds within the C-S-H gel. This peak sharpens with increasing curing age, indicating an increase in the degree of polymerization of the C-S-H gel [47]. This peak sharpens with increasing curing age, indicating an increase in the degree of polymerization of the C-S-H gel. These results are consistent with the XRD and SEM findings, which show an increase in AFt formation from 3 days to 7 days.

### 4.3. SEM Analysis

**Figure 8:**
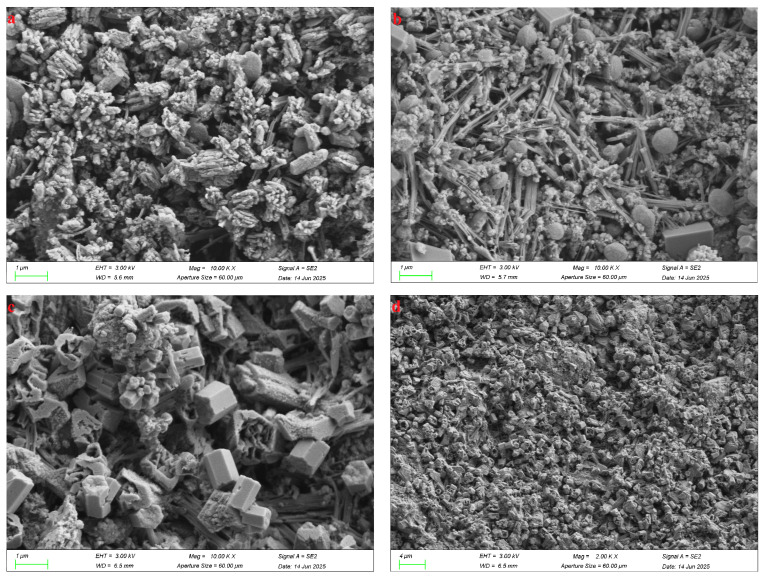
Micrographs of SZ-11 specimens cured for 3 d (**a**), 7 d (**b**) and 28 d (**c**,**d**).

Figure 8 presents the microstructural evolution of the SZ-11 specimen at three critical curing ages: 3 d, 7 d, and 28 d. Through XRD confirmation of the crystalline phase of the AFt phase and the FTIR spectral evidence from intensity variations in the Si–O–T stretching/bending vibrational bands at 1116 cm^−1^ and 463 cm^−1^ (indicating the polymerization degree of C–S–H), the SEM images further reveal, in high resolution, temporal disparities in the spatial distribution and morphological characteristics of hydration products.

The AFt crystals formed after 3 days progressively develop into coarse, rod-like morphologies over 28 days of hydration (Figure 8a). At 3 days, a high spatial density of AFt is evident within the specimen, a finding that corroborates the results obtained from X-ray diffraction, infrared spectroscopy, and thermogravimetric analysis. Owing to the limited degree of hydration, the SZ-11 sample exhibits poor compactness and pronounced interstitial porosity after 3 days of curing. With prolonged hydration up to 28 days, the microstructure becomes markedly denser (Figure 8c,d). This densification is attributed to the continuous precipitation of calcium–silicate–hydrate (C-S-H) gel and the concurrent growth of AFt crystals, which progressively infill the internal voids of the SZ-11 matrix and overlap the surfaces of unreacted particles. Consequently, the initially discrete solid phases become bridged, yielding a more compact, cohesive, and mechanically robust microstructure.

### 4.4. Thermogravimetric Analysis

**Figure 9:**
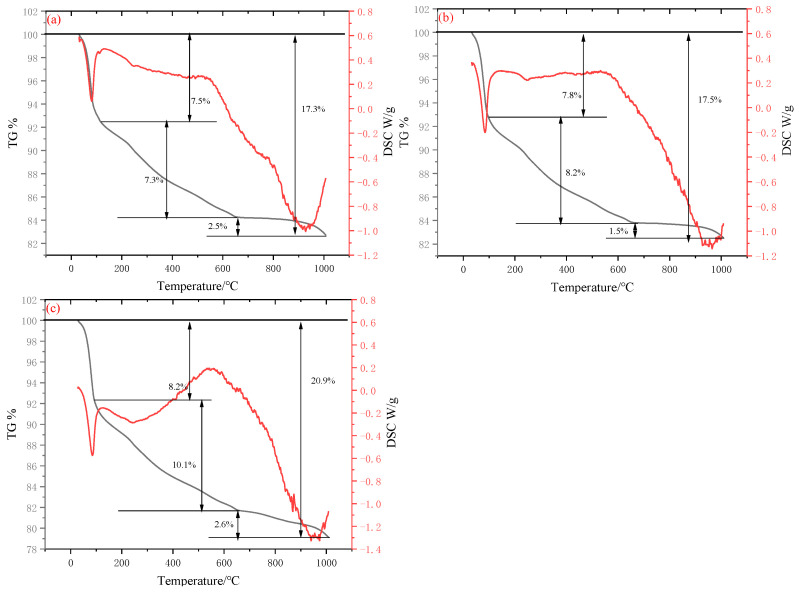
TG-DSC curves of hardened paste at various hydration ages ((**a**): 3 days; (**b**): 7 days; (**c**): 28 days).

As illustrated in Figure 9, the specimens exhibit three pronounced mass-loss regions at all investigated hydration ages, and the cumulative mass loss at 28 d consistently exceeds that at 3 d. The three regions are centred at 30–150 °C, 200–600 °C and 650–1000 °C.

Thermogravimetric (TG) analysis revealed that the cementitious material exhibited a characteristic stepwise mass loss pattern throughout the heating process. As the temperature increased progressively from room temperature, the first distinct weight loss step occurred within the temperature range of 30 °C to 150 °C. Concurrently, a prominent endothermic valley was observed in the Differential Scanning Calorimetry (DSC) curve over this same temperature interval. This behavior is primarily attributed to the evaporation of free water and the removal of weakly bound water, such as interlayer water molecules within C-S-H gel. This phenomenon represents a typical thermoresponsive signature associated with the initial hydration products of cementitious materials [48]. Subsequently, a second weight loss step can be observed between 200 °C and 600 °C. This stage is primarily attributed to the thermal decomposition of Ca(OH)_2_ [49]. The third weight loss step occurs in the range of 650 °C to 1000 °C. This stage primarily involves the final dehydration of both C-S-H gel and AFt [50]. The observed endothermic range around 850 °C in the curve may correspond to the crystallization of cementitious materials, as supported by the hydration heat data and the chemical reactions of cement components. As the amount of hydration products increases, both the internal structure and mechanical properties are enhanced. Alkaline substances in RM combine with Ca^2+^, forming calcium hydroxide that further reacts with active silica and alumina to generate gels such as AFt and C-S-H. Cementitious materials exhibit significant synergistic effects among RM, EMR, and SS, ultimately enhancing the hydration degree of the material.

It is noteworthy that as the total hydration products accumulate over time, the cumulative weight loss corresponding to each stage of TG increased from 17.3% at 3 days (10.27 MPa) to 20.9% at 28 days (20.48 MPa). This indicates a significant increase in chemically bound water content and densification of the internal microstructure. The alkaline components in RM (primarily NaOH and KOH) rapidly release OH^−^ during early hydration, reacting with dissolved Ca^2+^ to form Ca(OH)_2_. This further undergoes pozzolanic reactions with reactive SiO_2_ and Al_2_O_3_ from EMR and SS, continuously generating C-S-H and AFt gels. The synergistic effects among the three solid wastes manifest as: RM provides an alkaline environment and Al^3+^, EMR contributes soluble silicon and trace SO_4_^2−^, while SS supplements Ca^2+^. This forms a multi-reaction system of “alkaline activation-pozzolanic-sulfoaluminate”, significantly enhancing both the hydration degree and mechanical properties of the cementitious material.

### 4.5. Hydration Mechanism

**Figure 10:**
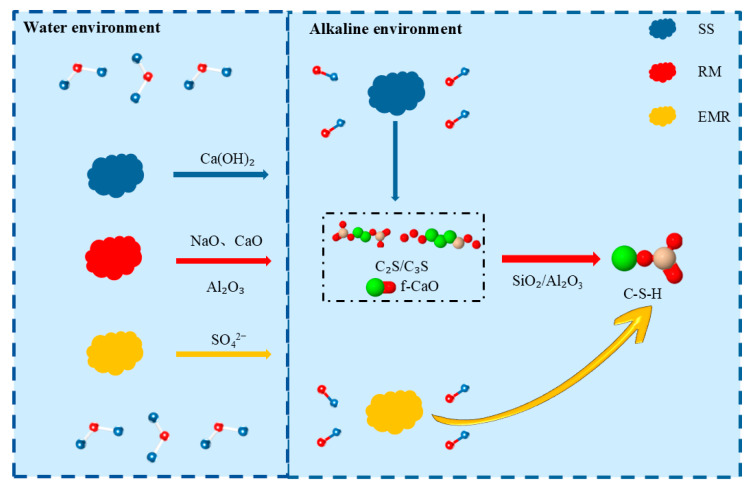
Mechanism of the electrolytic manganese residue–red mud–steel slag all-solid-waste cementitious system.

The hydration mechanism of the composite system is schematically illustrated in Figure 10. Red mud functions as an alkaline activator by rapidly releasing Na_2_O and CaO, thereby establishing a highly alkaline environment (pH > 13) that effectively activates the latent reactivity of both steel slag and electrolytic manganese residue. The Al_2_O_3_ present in red mud concurrently serves as an essential precursor for the subsequent formation of hydration products such as calcium aluminate hydrates. Steel slag constitutes the principal source of long-term strength development and alkalinity regulation within the system. The C_2_S and C_3_S phases are the dominant contributors to mechanical strength, whereas the progressive hydration of these phases continuously generates Ca(OH)_2_, which sustains the alkaline conditions required for pozzolanic reactions. The free CaO (f-CaO) contained in steel slag is rapidly consumed by the reactive SiO_2_ and Al_2_O_3_ supplied by red mud and electrolytic manganese residue under the prevailing alkaline conditions, thereby converting a potentially deleterious component into additional C-S-H gel. Electrolytic manganese residue functions as both a supplementary cementitious material and a micro-aggregate. Under an alkaline environment, the vitreous phase of manganese residue undergoes depolymerization and activation, resulting in the formation of supplementary C-S-H gel. The abundant SiO_2_ present in manganese residue serves as a key reactant for f-CaO consumption, thereby further promoting C-S-H precipitation. Additionally, the fine-grained fraction of electrolytic manganese residue effectively refines the pore structure by physical packing, thereby optimizing the overall microstructural density of the composite system.

This ternary system avoids the detrimental effects of “alkali excess” often induced by high red mud content, while simultaneously harnessing the inherent cementitious properties of steel slag. An optimal balance is achieved between alkaline activation efficiency and gel-forming contribution. The high alkalinity derived from red mud activates the pozzolanic/reactivity potential of both steel slag and electrolytic manganese residue. Meanwhile, the active Si–Al components provided by electrolytic manganese residue and red mud effectively consume the free CaO in steel slag, thereby preventing harmful expansion. Through combined chemical reactions and physical densification mechanisms among the three solid wastes, a cementitious system with robust strength development and enhanced volumetric stability is formed.

## 5. Conclusions

This study developed a novel ternary cementitious system entirely from industrial solid wastes—red mud (RM), steel slag (SS), and electrolytic manganese residue (EMR). The key findings are summarized as follows:

(1) The formulation incorporating 30% RM, 50% SS and 20% EMR achieved a 28-day compressive strength of 20.40 MPa at a water-glue ratio of 0.28, complying with ASTM C595. This ratio balances alkalinity and stability while maximizing hydration activity.
(2) Statistical analysis (ANOVA) identified RM content as the most significant factor (F = 6.788 > F critical = 4.760), highlighting the critical function of alkali in activating SS and EMR.
(3) RM establishes a high-pH environment, SS supplies Ca^2+^ and cementitious phases, and EMR contributes reactive SiO_2_/Al_2_O_3_ and micro-aggregate filling. The system converts harmful f-CaO from SS into additional C-S-H gel via reaction with silico-aluminates from RM and EMR, ensuring both high strength and volume stability.

The principal innovation of this work lies in the design of a fully solid-waste binder system that achieves efficient synergy in alkaline activation and volume stability through precisely balanced material proportions. It demonstrates a new pathway for high-value utilization of challenging industrial solid wastes and represents a materials design philosophy that transforms harmful properties into beneficial features through rational component coupling.

## Figures and Tables

**Table 1:** Chemical composition of raw materials %.

Component Content	SiO_2_	CaO	Al_2_O_3_	Fe_2_O_3_	NaO_2_	TiO_2_	K_2_O	MgO	SO_3_	MnO	P_2_O_5_	Others
EMR	26.95	7.82	6.27	3.74	0.75	0	1.58	2.09	22.94	2.27	0	24.68
RM	28.11	2.62	22.17	28.46	10.34	1.65	0.14	0.25	0	0	0	6.26
SS	11.74	42.82	10.64	20.31	0	1.02	0	5.33	0.75	3.85	1.41	2.13

**Table 2:** Toxicity Leaching Results (µg/L).

Component Content	Mn	As	Cd	Cr	Cu	Pb	Fe
EMR	1,245,000	0.39	11.8	<0.11	<0.08	<0.09	112
RM	223.9	148.6	0.17	184.7	1.7	<0.09	133.9
SS	318.8	0.14	<0.05	<0.11	0.93	<0.09	237

**Table 3:** Blend ratios of different raw materials.

Name	RM	SS	EMR	Water-to-Binder Ratios
S1	60%	30%	10%	0.31
S2	50%	35%	15%	0.31
S3	40%	40%	20%	0.31
S4	30%	45%	25%	0.31

**Table 4:** Different Water-Binder Ratio Test Table.

Name	Water-to-Binder Ratios	RM:SS:EMR
A-1	0.28	2:2:1
A-2	0.30	2:2:1
A-3	0.32	2:2:1
A-4	0.34	2:2:1
A-5	0.36	2:2:1

**Table 5:** Orthogonal factor table.

Factor			
Level	RM	EMR	Water-to-Binder Ratios
1	60%	10%	0.28
2	50%	15%	0.30
3	40%	20%	0.32
4	30%	25%	0.34

**Table 6:** Orthogonal experimental table.

	RM	EMR	Water-to-Binder Ratios
SZ-1	60%	10%	0.28
SZ-2	60%	15%	0.30
SZ-3	60%	20%	0.32
SZ-4	60%	25%	0.34
SZ-5	50%	10%	0.30
SZ-6	50%	15%	0.28
SZ-7	50%	20%	0.34
SZ-8	50%	25%	0.32
SZ-9	40%	10%	0.32
SZ-10	40%	15%	0.34
SZ-11	40%	20%	0.28
SZ-12	40%	25%	0.30
SZ-13	30%	10%	0.34
SZ-14	30%	15%	0.32
SZ-15	30%	20%	0.30
SZ-16	30%	25%	0.28

**Table 7:** The Range Analysis Table.

	RM	EMR	Water-Glue Ratio
average value 1	8.90	11.125	14.85
average value 2	10.70	11.625	12.825
average value 3	14.925	13.60	11.225
average value 4	15.4	13.575	11.025
range (R)	6.5	2.475	3.825

**Table 8:** Analysis of Variance Table. (*, this symbol stands for significance.)

Factor	Deviation Sum of Squares	Degree of Freedom	F-Value	F-Critical Value	Significance
RM	121.957	3	6.788	4.760	*
EMR	20.082	3	1.118	4.760	
Water-glue ratio	37.712	3	2.099	4.760	
error	35.93	6			

**Table 9:** Verification Test Compressive Strength Test Table (MPa).

Name	3 d	7 d	28 d
1	10.25	11.95	20.54
2	10.30	11.90	20.43
3	10.26	12.00	20.51
mean	10.27	11.95	20.48
orthogonal test results	10.20	12.00	20.40

**Table 10:** Cement-based cementitious materials and solid waste cementitious materials compressive strength results (MPa).

Group	Name	3 d	7 d	28 d
1	cement-based gelling material	30.5	36.4	49.6
2	solid waste based cementitious material	10.2	12.0	20.4

## Data Availability

The original contributions presented in this study are included in the article. Further inquiries can be directed to the corresponding author.

## Abbreviations

The following abbreviations are used in this manuscript:

RM	Red mud
EMR	Electrolytic manganese residue
SS	Steel slag
C-S-H	Hydrate calcium silicate
AFt	Ettringite
f-CaO	Free-CaO
